# A pilot optical coherence tomography angiography classification of retinal neovascularization in retinopathy of prematurity

**DOI:** 10.1038/s41598-023-49964-8

**Published:** 2024-01-04

**Authors:** Xi Chen, Ryan Imperio, Christian Viehland, Pujan R. Patel, Du Tran-Viet, Shwetha Mangalesh, S. Grace Prakalapakorn, Sharon F. Freedman, Joseph A. Izatt, Cynthia A. Toth, BabySTEPS Group

**Affiliations:** 1 Department of Ophthalmology, Duke University Medical Center, Durham, NC 27517 USA; 2 Department of Pediatrics, Duke University Medical Center, Durham, NC 27571 USA; 3 Department of Biomedical Engineering, Duke University, Durham, NC 27517 USA; 4 Duke University Medical Center, 2351 Erwin Road, Box 3802, Durham, NC 27710 USA

**Keywords:** Retinopathy of prematurity, Retinal diseases

## Abstract

Extraretinal neovascularization is a hallmark of treatment-requiring retinopathy of prematurity (ROP). Optical coherence tomography angiography (OCTA) offers vascular flow and depth information not available from indirect ophthalmoscopy and structural OCT, but OCTA is only commercially available as a tabletop device. In this study, we used an investigational handheld OCTA device to study the vascular flow in and around retinal neovascularization in seven preterm infants with treatment-requiring ROP and contrasted them to images of vascular flow in six infants of similar age without neovascular ROP. We showed stages of retinal neovascularization visible in preterm infants from 32 to 47 weeks postmenstrual age: Intraretinal neovascularization did not break through the internal limiting membrane; Subclinical neovascular buds arose from retinal vasculature with active flow through the internal limiting membrane; Flat neovascularization in aggressive ROP assumed a low-lying configuration compared to elevated extraretinal neovascular plaques; Regressed neovascularization following treatment exhibited decreased vascular flow within the preretinal tissue, but flow persisted in segments of retinal vessels elevated from their original intraretinal location. These findings enable a pilot classification of retinal neovascularization in eyes with ROP using OCTA, and may be helpful in detailed monitoring of disease progression, treatment response and predicting reactivation.

## Introduction

Retinal neovascularization is a hallmark of advanced retinopathy of prematurity (ROP). It is caused by the combination of immature or delayed retinal vascular growth and an overproduction of vascular endothelial growth factor produced by the avascular retina^1^. The neovascularization is sometimes difficult to identify and document, therefore, fluorescein angiography has been used to highlight with leakage and aid in the characterization of the presence, location, and morphology of retinal neovascularization in ROP, as well as defining the vascular-avascular junction^2–4^. Handheld optical coherence tomography (OCT) adds depth information and allows additional three-dimensional reconstruction of the structure of these neovascular tissues, yet it does not allow detection or characterization of retinal vascular flow^4–6^. The advent of handheld OCT angiography (OCTA) has facilitated non-invasive examination of the depth-resolved capillary flow in infants without intravenous dye injection, and has been recently adapted for handheld use in research studies^7–14^. These investigational devices provide an opportunity to study in detail the neovascular flow in ROP.

Our group has previously reported the stages of neovascularization on three-dimensional structural OCT as neovascular buds, bridging network, and placoid lesions^6^. There have also been reports of retinal neovascular flow using commercial tabletop OCTA devices and enhanced visualization using an investigational handheld OCTA system^10–12^. However, there has not been a detailed OCTA classification of neovascularization in ROP to date.

As part of the Study of Eye imaging in Preterm infantS (BabySTEPS, NCT02887157)^9^, we used an investigational handheld noncontact 200 kHz swept-source OCTA system at the bedside to capture the capillary flow within retinal neovascular tissues in infants with severe ROP and within the developing vessels of those without severe ROP. In contrast to imaging physiological vascularization in preterm infants, we characterize stages of retinal neovascularization including intraretinal neovascularization, extraretinal neovascular buds, and extraretinal neovascular plaques. Additionally, we describe extraretinal flat neovascularization in aggressive ROP and regressed extraretinal neovascularization following treatment.

## Results

**Figure 1:**
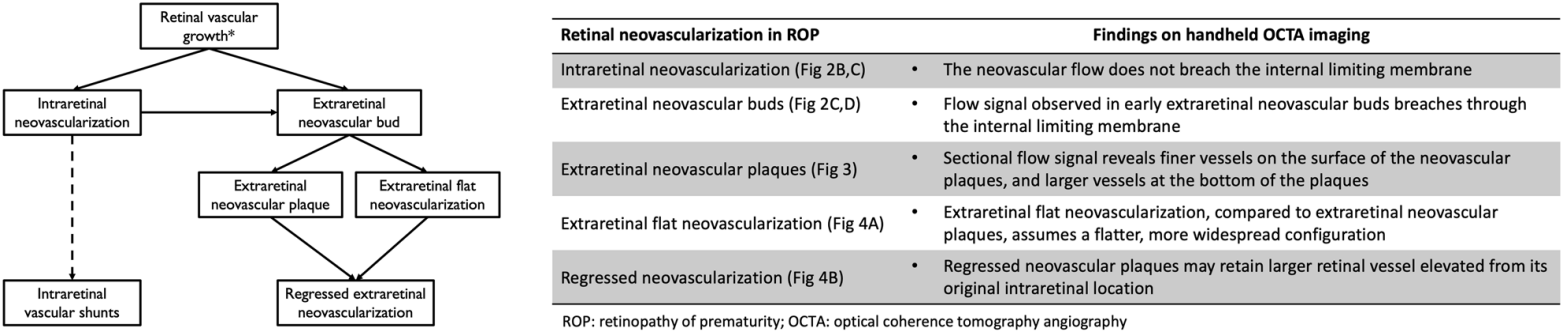
Pilot Classification of the development and sequelae of intra- and extraretinal neovascularization in retinopathy of prematurity using optical coherence tomography angiography.

We included OCTA images of good quality from 14 eyes of 13 preterm infants enrolled in the prospective BabySTEPS imaged between 32 and 49 weeks post-menstrual age (PMA). We included 8 eyes of 7 preterm infants with or who subsequently developed treatment-requiring ROP (TR-ROP group; imaged at 32–47 weeks PMA). We compared them to images obtained from 6 eyes of 6 preterm infants without treatment-requiring ROP (non-TR-ROP group, without neovascularization or shunts; imaged at 36–49 weeks PMA). The infants’ demographic information and clinical characteristics are summarized in Supplementary Table 1. We included images with adequate quality documenting vascular flow from 15 imaging sessions. The OCTA findings and proposed stages of retinal neovascularization in ROP are summarized in Fig. 1.

### Retinal vascular axial distribution and pathological intraretinal neovascularization

**Figure 2:**
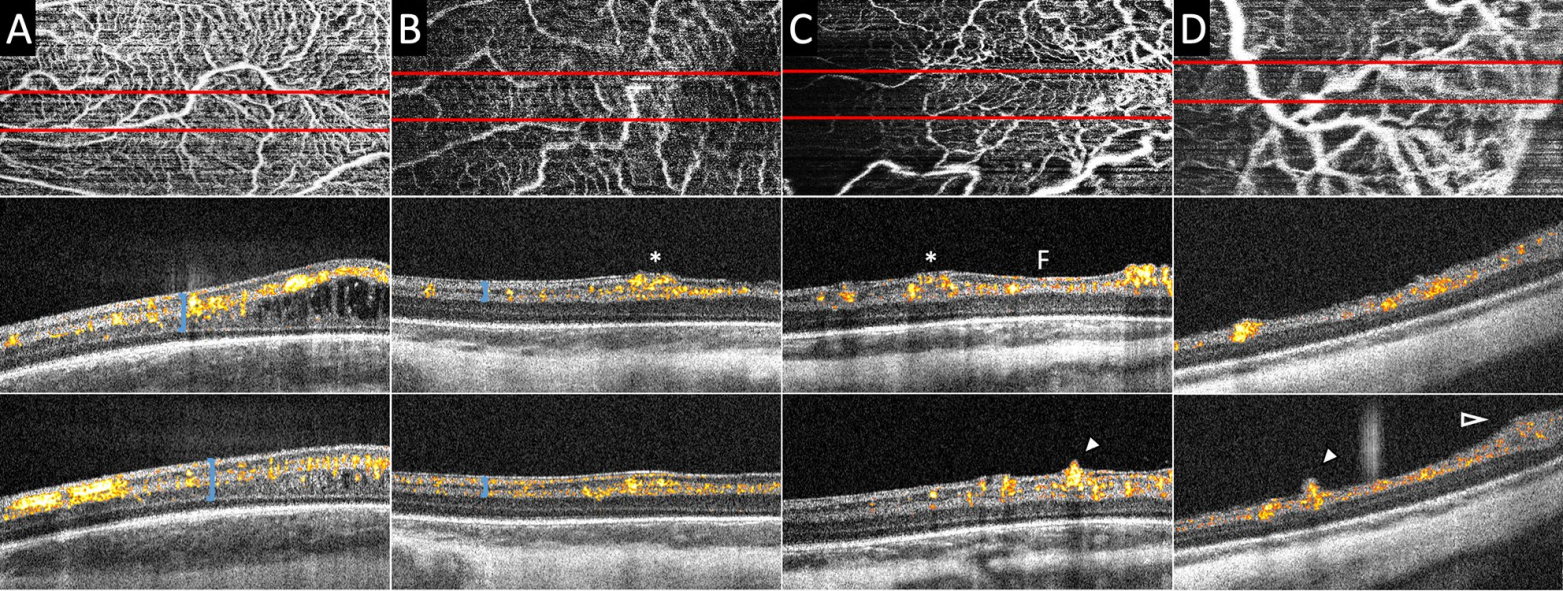
Bedside handheld optical coherence tomography angiography (OCTA) images of the preterm infant retinal vasculature in eyes without (**A**) and with (**B** and **C**, same infant, &**D**) neovascularization. The top row shows the vascular pattern en face, the bottom two rows are cross-sectional B-scans with flow overlay from the top row images at the site of the red lines. OCTA flow signal appears yellow and red in cross-section and sums to create the white vascular pattern of the en face images in the top row. (**A**) At 36 weeks postmenstrual age (PMA) in an infant who never developed severe ROP, retinal microvasculature was well-formed in the macula with flow signal present from the innermost border of the retina (internal limiting membrane) to the inner nuclear layer/inner plexiform layer junction. Macular edema was visible and common in preterm infants. (**B**) At 32 weeks PMA in an infant who later required treatment for severe ROP, prominent vascular flow was observed in the perifoveal region, with focal mild elevation of the inner retinal surface (asterisk). (**C**) Two weeks later (at 34 weeks PMA), the same eye as in (**B**) developed severe (aggressive) ROP that required treatment. The inner retinal neovascularization produced greater elevation and splitting of the internal limiting membrane / nerve fiber layer on either side of the fovea (**F**), and now breached the internal limiting membrane in the inferior macula (arrowhead), assuming an early extraretinal neovascular bud configuration. (**D**) At 34 weeks in an infant who required treatment of severe ROP one week later, an extraretinal neovascular bud (arrowhead) exhibited flow signal extending above the inner retinal surface over a larger retinal vessel posterior to the margin of vascularized retina. Neovascular flow signals also extended into the preretinal tissue at the vascular-avascular junction (open arrowhead).

In the six eyes in the non-TR-ROP group imaged between 36 and 49 weeks PMA (with clinical ROP status ranged from stage 0–2 at the time of imaging), retinal microvascular flow was axially distributed from within the retinal nerve fiber layer, ganglion cell layer, inner plexiform layer, and the inner nuclear layer (INL) to the outer margin of the INL on the B-scans with flow overlay (bracket, Fig. 2A). In the eyes in the TR-ROP group imaged between 32 and 47 weeks PMA (with clinical ROP status ranged from stage 0–3 and aggressive ROP at the time of imaging), the vascular flow signal was predominantly superficial to INL, without obvious flow signal observed in or below the inner margin of the INL at the time of imaging (blue bracket in Fig. 2B in comparison to Fig. 2A).

In one eye with aggressive ROP at 34 weeks PMA, vessels with prominent flow signal were found on either side of the fovea (marked with “F”, Fig. 2C, middle panel). A similar, but less robust vascular structure with flow signal was observed at the same location 2 weeks prior (at 32 weeks PMA, at which time with stage 0 ROP on clinical exam, asterisks marking the same location, Fig. 2B middle panel). The observed intraretinal neovascularization appeared to be clusters of fine vascular flow signal with their top elevating from the retinal surface, but did not breach the internal limiting membrane. There was an exception at one location in the inferior macula, where the internal limiting membrane was breached and the neovascular tissue assumed the configuration of an early extraretinal neovascular bud (Fig. 2C, arrowhead).

### Extraretinal neovascular buds

In four eyes in the TR-ROP group with clinical stage 2–3 ROP, extraretinal neovascular buds arose from the retinal vasculature and breached the internal limiting membrane with active vascular flow signal on OCTA. The buds could be seen isolated at the posterior pole, or near the vascular-avascular junction. The flow signal was continuous and appeared to stem from a larger retinal vessel located in the ganglion cell layer-inner plexiform layer (Fig. 2D, arrowhead).

### Extraretinal neovascular plaques

**Figure 3:**
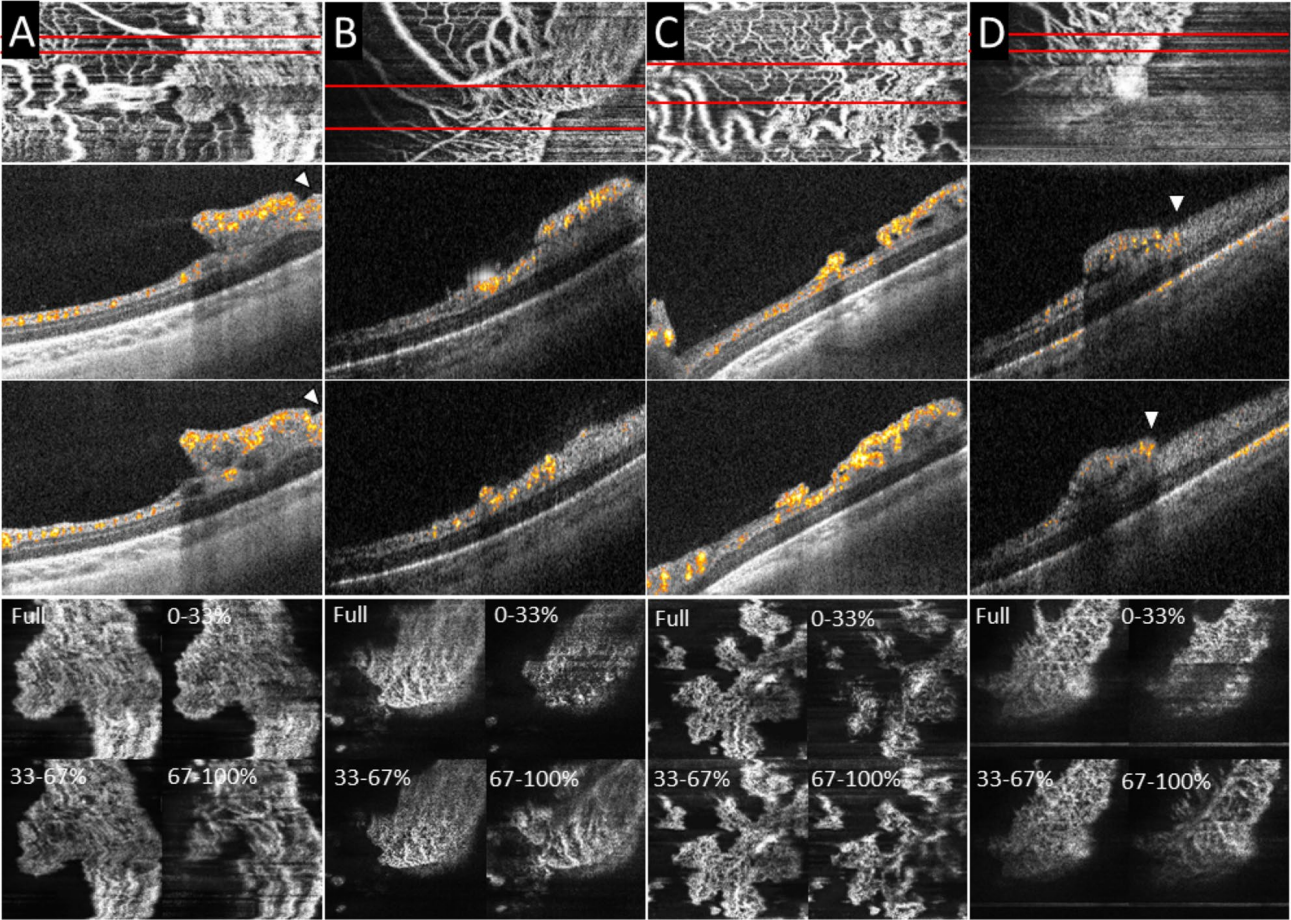
Extraretinal neovascular plaques and distribution of vascular flow by axial location within the neovascular plaques on handheld optical coherence tomography angiography (OCTA). Top row: en face OCTA flow; Second and third rows: B-scans with flow overlay; Bottom row: en face sub-sectional flow (generated by the percentage of distance from the top to bottom of the neovascular plaque). In three eyes with treatment requiring-ROP, extraretinal neovascular plaques were found elevated from the retinal surface along the vascular-avascular junction. On OCT B-scans with flow overlay, the retinal vascular flow signal extends to the edge or beyond the edge of the neovascular plaque (arrowheads). The vascular flow signal on cross-section on B-scans with flow overlay and the sub-sectional flow analysis (Full, 0–33%, 33–67%, and 67–100%) showed finer capillaries at the surface of the plaque (vitreous side) and larger, more dilated capillaries at the bottom of the plaque (retina side).

In four eyes in the TR-ROP group with clinical stage 3 ROP, extraretinal neovascular plaques were found breaching the internal limiting membrane and rising above the retinal surface at the vascular-avascular junction, assuming an elevated, stuck-on appearance (Fig. 3). Sub-sectional flow analysis showed that within the neovascular plaques, thinner capillaries appeared in the upper third, vitreous side of the neovascular plaque (0–33% in flow analysis panel), while larger, more dilated capillaries were found at the bottom third, retina side of the plaque (67–100% on flow analysis panels). The retinal vascular flow signals extended to the edge or just beyond the extent of the neovascular plaques (Fig. 3, arrowheads). While most plaques appeared to be coalesced neovascular tissue stemming from multiple larger retinal vessels, we observed fine vascular flow in heaped-up inner retinal thickening at the vascular-avascular junction in an infant eye with clinical appearance of stage 2 ROP (Fig. 2D bottom panel, open arrowhead).

### Extraretinal flat neovascularization in aggressive ROP

**Figure 4:**
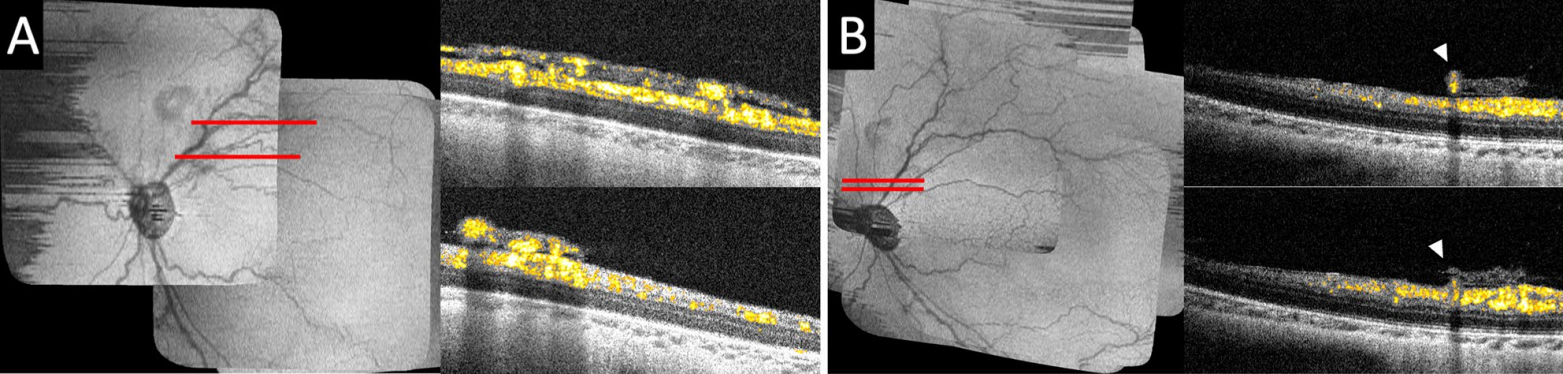
Optical coherence tomography angiography of extraretinal flat neovascularization in an eye with aggressive retinopathy of prematurity (ROP), and regressed extraretinal neovascularization following intraretinal bevacizumab treatment. (**A**) In an eye with aggressive ROP at 36 weeks PMA, OCT B-scans with flow overlay near the posterior pole superior to the optic disc along the superotemporal arcade showed vascular flow in the extraretinal neovascular tissue appeared flatter and over a wider retinal surface area compared to the extraretinal neovascular plaques. (**B**) The same eye was imaged at 47 weeks PMA following bevacizumab treatment at 36 weeks PMA. The preretinal neovascular tissue was regressed and marked reduction of vascular flow within the preretinal tissue. However, one larger retinal vessel was seen (arrowheads) retained in the preretinal tissue elevated from its original intraretinal location.

In an eye with aggressive ROP at 36 weeks PMA, extraretinal neovascular tissue was found in the posterior pole and assumed a flatter configuration over a wider surface area (Fig. 4A) compared to typical extraretinal neovascular plaques (Fig. 3). It appeared to be a coalescence of multiple extraretinal neovascular buds just superior to the optic disc. This extraretinal neovascular flow corresponded to what was clinically described as flat neovascularization observed in infants with aggressive ROP. Compared to the extraretinal neovascular plaques in which flow signal was seen in multiple layers stemming from one or few peripheral feeder vessels, flow signal could be seen more diffusely across the extraretinal neovascular tissue, with multiple distinct stems arising from the underlying superficial retinal vasculature.

### Regressed extraretinal neovascularization

At 36 weeks PMA, the eye with extraretinal flat neovascularization received intravitreal bevacizumab treatment. Eleven weeks after both this imaging and bevacizumab treatment, at 47 weeks PMA with clinical regressed ROP, regression of the extraretinal neovascularization and significantly decreased vascular flow within the preretinal tissue was evident on OCTA (Fig. 4B). The overall size of the preretinal tissue was reduced and assumed a wispy appearance. It is notable that nearly 3 months after the treatment, flow was visible in an abnormally elevated preretinal segment of a larger retinal vessel retained in the regressed preretinal tissue (Fig. 4B, arrowheads). This was not appreciated by clinical examination.

We also observed in infants in the TR-ROP group vascular dilation and tortuosity on en face OCTA images (visible in Figs. 2D and 3C) and vascular loops (visible in Fig. 2D at the vascular-avascular junction). We did not, however, observe any loss of vascular flow in areas leading to the neovascular tissues.

## Discussion

Our current study uses handheld OCTA in preterm infants to characterize and classify retinal neovascularization in eyes with severe ROP compared to eyes without neovascularization. Our observations highlight the use of handheld OCTA images to differentiate and classify intraretinal versus extraretinal neovascularization in ROP, and the location of the retinal microvasculature by depth in normally and abnormally developing retinas of preterm infants.

Retinal neovascularization is traditionally imaged with fluorescein angiography, and has only more recently, been visualized by OCTA. The recent advancement in wide-field tabletop OCTA technology has enabled imaging of non-central neovascularization in adults^15^, in diseases such as proliferative diabetic retinopathy^16–21^, macular telangiectasia^22^, and type 3 neovascularization of age-related macular degeneration^23^. However, the current tabletop systems are not suitable for imaging awake, supine infants who are unable to cooperate with positioning or fixation. OCTA, with its noninvasive nature and depth resolution, is a promising method to repeatedly image and analyze vascular growth and the detailed anatomy of retinal neovascularization in ROP. Applying OCTA imaging through a handheld device at the infant bedside has been facilitated by the increased speed of swept-source OCT image capture and decrease in weight of the investigational device as in this study^9^. In addition, the en face view of the handheld OCTA was superior in determining the exact location of the retinal vascular-avascular junction in preterm infants compared to handheld structural OCT. In our prior study, the vascular-avascular junction was identified through comparison of the retinal vascular projection on OCT retina view and structural OCT, and with this visualization, the vascular-avascular junction appeared located within the retina covered by the neovascular plaque^24^. In our current study, using OCTA imaging which is more sensitive to vascular flow within the retinal tissue, retinal vascular flow signals are visible at the anterior edge or just beyond the extent of the neovascular plaques.

We found that the vascular morphology of neovascular plaques in infants with ROP may be distinct from retinal neovascularization in adult patients with proliferative diabetic retinopathy. Two morphological categories of the neovascularization elsewhere have been reported in diabetic retinopathy, a round type and a ramified type, both with vascular structures of a similar vessel caliber^21^. In contrast, neovascular plaque in ROP appeared to have a more complex capillary structure, with smaller capillaries on the top of the neovascular lesion and larger vessels at the base, closer to the retina. This may be due to neovascular growth from the immature, developing retinal vessels in ROP, rather than the mature but injured retinal vessels in diabetic retinopathy. The neovascular plaques in ROP also appeared to be thicker with enlarged neovascular stems rather than thinner, sparser stems of neovascularization in diabetes. These morphological differences may enlighten us on the differences in pathophysiology of neovascularization in ROP versus proliferative retinopathy in adulthood.

Aggressive ROP is a rapidly progressing, severe form of ROP that is characterized by posterior location, prominence of plus disease out of proportion to classic peripheral disease, and without stepwise progression through classic stages^25,26^. Associated vascular changes include flat neovascularization and shunt vessels. In a preterm infant eye with aggressive ROP following initial laser treatment, OCTA imaging was obtained in an infant swaddled and held upright in the chin rest with the Avanti RTVue OCT^10^. In that infant, flow signal was seen above and within an area in the mid-peripheral retina and it was hypothesized that the flat neovascularization and its deeper extension constitute a neovascular complex. In our current study, we observed flat neovascularization to have a flat configuration with multiple stems arising from the underlying larger retinal vasculature, which occupied a larger feeding retinal area compared to the extraretinal neovascular plaques found near the vascular-avascular junction. Interestingly, following regression of the flat neovascularization after treatment, a segment of the larger retinal vessel was noted to be “left behind” in the now regressed neovascular tissue without other significant neovascular flow. Future longitudinal studies will allow for analysis of the development and longitudinal progression of these changes.

Handheld OCTA may help identify precursor lesions prior to breakthrough of the internal limiting membrane. We observed neovascular changes in two eyes before ROP treatment: we detected perifoveal neovascularization in one eye and posterior neovascular buds in another eye 2 weeks before they were treated with intravitreal bevacizumab. Perifoveal neovascularization is not uncommon in infants with severe ROP. It has also been reported in proliferative diabetic retinopathy and macular telangiectasia type 2^22,27^. The perifoveal neovascularization may be due to the relative transition from vascularized to avascular retina near the fovea, and/or its underlying oxygen or VEGF gradient. It will be worthwhile to investigate if these findings of posterior neovascular activity will aid in prediction and early identification of TR-ROP.

Interestingly, we did not observe superficial capillary bed loss or vascular flow void posterior to neovascular plaques as previously seen on fluorescein angiography or in mouse models of ROP^28^. This previously observed capillary bed loss could be due to the hyperflow in the nearby neovascular lesions on fluorescein angiography masking the physiological blood flow as seen on OCTA. In addition, compared to the axial distribution of vessels in infant retinas without severe ROP, we did not observe obvious flow signal in the deeper vascular complex in the areas of neovascularization. This finding may be due to the delayed deep vascular complex development in infants with TR-ROP. However, the lack of flow signal could also be due to the much larger vascular flow in the superficial and extraretinal neovascular tissue shadowing over the deeper retina and warrants additional investigation.

Our current study is limited by small sample size and inconsistent longitudinal data capture due to difficulty of performing handheld OCTA in awake infants. OCTA image capture could potentially be improved with higher speed engine and/or a pupil camera to help with alignment to capture images. Nonetheless, we report the capture of the structure and depth-resolved capillary flow of retinal neovascular tissue with an innovative high-speed investigational handheld noncontact swept source OCTA system at the bedside. These initial findings of retinal neovascular flow in awake infants with ROP advance our understanding of retinal neovascularization in infants due to ROP. Future use of this technology may be helpful in monitoring disease progression, detailing treatment response and vascular remodeling, and predicting reactivation of ROP.

## Methods

### Subjects

This study is a secondary analysis of the OCTA imaging component of the prospective BabySTEPS infant retinal imaging study (clinicaltrial.gov: NCT02887157), which was approved by the Duke University Health System Institutional Review Board and adhered to the Health Insurance Portability and Accountability Act and all tenets of the Declaration of Helsinki. Premature infants undergoing ROP screening at Duke University Hospital was enrolled with written informed consent from a parent/legal guardian. Infants with severe neurological or ocular comorbidities were excluded.

### OCT and OCTA imaging

OCT and OCTA images were acquired at the bedside in the neonatal intensive care nursery or transitional care nursery using an investigational 200 kHz swept-source handheld OCT device (UC3, Duke University, Durham, NC)^9^. Investigational OCTA imaging was performed when there was time allowed following research structural OCT imaging as described separately^29^. Volumes with adequate image quality were captured at 500 A-scans per B-scan and 250 B-scans per volume repeated four times at each lateral location (scan size equivalent to 2.5 × 5 mm in adult eyes). The image acquisition time was approximately 2.4 s per OCTA volume.

OCTA imaging volumes were processed with custom software (Duke OCT Retinal Analysis Program (DOCTRAP) 65.6) (MATLAB R2017b, MathWorks, Natick, MA) optimized for infant retinal image processing and preliminary OCTA processing using MATLAB scripts. Quality image volumes were selected by experienced infant OCTA graders (RI and XC). Retinal layers on each individual B-scan were first automatically segmented using a custom software. The images were then reviewed and manually corrected by infant OCTA graders (RI and XC) and preliminary processing was performed. The image selection was based on volumes with clear visualization of retinal vascular patterns and few motion artifacts. Images were then processed with line-based correction, histogram equalization and contrast adjusted with custom MATLAB scripts. B-scans with flow overlay were then generated with custom MATLAB scripts^29^. Images documenting neovascularization in ROP were first selected for study inclusion (8 eyes of 7 infants), then age-matched controls were selected in eyes that never developed neovascular ROP (6 eyes of 6 infants).

### Supplementary Information

Supplementary Information 1.

Supplementary Table S1.

## Data Availability

The datasets generated during and/or analyzed during the current study are available from the corresponding author on reasonable request.

## References

[1] Hartnett ME, Penn JS (2012). Mechanisms and management of retinopathy of prematurity. N. Engl. J. Med..

[2] Klufas MA (2015). Influence of fluorescein angiography on the diagnosis and management of retinopathy of prematurity. Ophthalmology.

[3] Lepore D (2021). Early angiographic signs of retinopathy of prematurity requiring treatment. Eye (Lond).

[4] Lepore D (2011). Atlas of fluorescein angiographic findings in eyes undergoing laser for retinopathy of prematurity. Ophthalmology.

[5] Maldonado RS, Toth CA (2013). Optical coherence tomography in retinopathy of prematurity: Looking beyond the vessels. Clin. Perinatol..

[6] Mangalesh S (2019). Three-dimensional pattern of extraretinal neovascular development in retinopathy of prematurity. Graefes Arch. Clin. Exp. Ophthalmol..

[7] Campbell JP (2017). Handheld optical coherence tomography angiography and ultra-wide-field optical coherence tomography in retinopathy of prematurity. JAMA Ophthalmol..

[8] Moshiri Y (2019). Handheld swept-source optical coherence tomography with angiography in awake premature neonates. Quant. Imaging Med. Surg..

[9] Viehland C (2019). Ergonomic handheld OCT angiography probe optimized for pediatric and supine imaging. Biomed. Opt. Express.

[10] Vinekar A, Chidambara L, Jayadev C, Sivakumar M, Webers CA, Shetty B (2016). Monitoring neovascularization in aggressive posterior retinopathy of prematurity using optical coherence tomography angiography. J. AAPOS.

[11] Ni S (2021). High-speed and widefield handheld swept-source OCT angiography with a VCSEL light source. Biomed. Opt. Express.

[12] Nguyen TP (2021). Advantages of widefield optical coherence tomography in the diagnosis of retinopathy of prematurity. Front. Pediatr..

[13] Zhou K (2020). Quantitative handheld swept-source optical coherence tomography angiography in awake preterm and full-term infants. Transl. Vis. Sci. Technol..

[14] Nguyen TP (2022). Widefield optical coherence tomography in pediatric retina: A case series of intraoperative applications using a prototype handheld device. Front. Med..

[15] Russell JF (2019). Distribution of diabetic neovascularization on ultra-widefield fluorescein angiography and on simulated widefield OCT angiography. Am. J. Ophthalmol..

[16] Arya M (2019). Distinguishing intraretinal microvascular abnormalities from retinal neovascularization using optical coherence tomography angiography. Retina.

[17] Russell JF (2019). Longitudinal wide-field swept-source OCT angiography of neovascularization in proliferative diabetic retinopathy after panretinal photocoagulation. Ophthalmol. Retina.

[18] Schwartz R (2020). Objective evaluation of proliferative diabetic retinopathy using OCT. Ophthalmol. Retina.

[19] Lu ES (2022). Detection of neovascularisation in the vitreoretinal interface slab using widefield swept-source optical coherence tomography angiography in diabetic retinopathy. Br. J. Ophthalmol..

[20] Pan J (2018). Characteristics of Neovascularization in early stages of proliferative diabetic retinopathy by optical coherence tomography angiography. Am. J. Ophthalmol..

[21] Shiraki A (2022). Analysis of progressive neovascularization in diabetic retinopathy using Widefield OCT angiography. Ophthalmol. Retina.

[22] Ayachit AG, Reddy LU, Joshi S, Ayachit GS (2019). Epiretinal neovascularization: A novel OCT angiography finding in macular telangiectasia type 2. Ophthalmol. Retina.

[23] Sacconi R (2018). Nascent type 3 neovascularization in age-related macular degeneration. Ophthalmol. Retina.

[24] Chen X (2018). Spectral-domain OCT findings of retinal vascular-avascular junction in infants with retinopathy of prematurity. Ophthalmol. Retina.

[25] International Committee for the Classification of Retinopathy of Prematurity (2005). The international classification of retinopathy of prematurity revisited. Arch. Ophthalmol..

[26] Vinekar A, Trese MT, Capone A, Photographic Screening for Retinopathy of Prematurity Cooperative Group (2008). Evolution of retinal detachment in posterior retinopathy of prematurity: Impact on treatment approach. Am. J. Ophthalmol..

[27] Murakawa S, Hasegawa T, Koizumi H, Maruko I, Iida T (2017). Foveal retinal neovascularization in proliferative diabetic retinopathy: Assessment by optical coherence tomography angiography. Retina.

[28] Yokoi T (2009). Vascular abnormalities in aggressive posterior retinopathy of prematurity detected by fluorescein angiography. Ophthalmology.

[29] Patel PR (2021). Depth-resolved visualization of perifoveal retinal vasculature in preterm infants using handheld optical coherence tomography angiography. Transl. Vis. Sci. Technol..

