# Supplementary material for: A pilot optical coherence tomography angiography classification of retinal neovascularization in retinopathy of prematurity
Source: Sci Rep. 2024 Jan 4;14:568. doi: 10.1038/s41598-023-49964-8 (PMC10766630; doi:10.1038/s41598-023-49964-8)
Supplement: Supplementary file 1 — Supplementary Information 1. [file 41598_2023_49964_MOESM1_ESM.pdf]

BabySTEPS Group
(Analysis of Retinal Microanatomy in Retinopathy of Prematurity to Improve Care Study Group)

Duke University:
Principal Investigator: Cynthia A. Toth, MD
Co-investigators: Xi Chen, MD, PhD; Charles M. Cotten, MD; Maysantoine El-Dairi, MD; Sina Farsiu, PhD; Sharon F. Freedman, MD; Hesham Gabr, MD; Sara Grace, MD; Kathryn E. Gustafson, PhD; Majda Hadziahmetovic, MD; Joseph Izatt, PhD; Ramiro Maldonado, MD; Suzanne Michalak, MD; Matthew O'Sullivan, MD, PhD; Sally Ong, MD; Miroslav Pajik, PhD; Carolyn Pizoli, MD; S. Grace Prakalapakorn, MD; Joan Roberts, MD; Rolake Alabi, MD, PhD; Nita Valikodath, MD; Lejla Vajzovic, MD; Christian Viehland, PhD; David Wallace, MD; Xiao Yi Zhou, MD
Research Scholars: Shwetha Mangalesh, MBBS; Vahid Ownagh, MD
Senior Research Program Lead: Michelle McCall, BA, MCAPM
Research Program Leads: Joanne Finkle, RN, JD; Neeru Sarin, MBBS
OCT Imagers: Alexandria Dandridge; Ryan Imperio, BS; William Raynor, BS; Du Tran-Viet, BS
Data Analysts: Stephanie Chiu, PhD; Heena Divecha, MS; Bo Hansen, MSE; Vincent Tai, MSE; Katrina P. Winter, BS
NPRU Director: Kimberly Fisher, PhD, FMP-BC
Coordinators: Lacey Andrews, BS; Melissa Babilonia-Rosa, PhD; Anne Baez Love, BS; Lucy DeStefano-Pearce, MA; Jessicka Hamilton, BA; Grace Jefferson, MS; Amanda Marion, BS; Isabella Pallotto, MPH; Marito Passero, BS; Caitlin Stone, MA; Michelle Sunico, BS
Undergraduate Students: Caelan Eckard, BS; Karthik Ganesan, BS; Xiao Tang, MS; Kira Wang, BA; Brittany Wong, BS
Graduate Students: Mark Draelos, MD, PhD; Francesco LaRocca, PhD; Amit Narewane, BSE; Qitong Gao, MS
Medical Students: Isaac Bleicher, MD; Pujan Patel, MD; Jay Rathinavelu, BSE; Kai Seely, MD, MHS; Mason Seely, BS

University of Pennsylvania:
Principal Investigator: Maureen G. Maguire, PhD (3/2016 – 2/2021); Gui-Shuang Ying, PhD (5/2021 – present)
Co-Investigator: Gui-Shuang Ying, PhD (3/2016 – 2/2021)
Statisticians: Brendan McGeehan, MS; Jocelyn He, MS

Washington University:
Principal Investigator: Joshua Shimony, MD, PhD
Other study personnel: Dimitrios Alexopoulos, MS; Sydney Kaplan, BS; Jeanette Kenley, BS; Kayla Hannon, BS

Data Safety and Monitoring Committee:
Chair: P. Brian Smith, MD, MPH, MHS, Duke University (6/2016 – present)
DSMC Members: Michael O'Shea, MD, MPH, University of North Carolina (7/2016 – 6/2021); Subashri Kurgatt, PhD, Duke University (7/2016 – 11/2020); Daniel X. Hammer, PhD, Food and Drug Administration (5/2021 – present); William Good, MD, Smith Kettlewell Eye Institute (5/2021 – present)
