# Supplementary material for: A pilot optical coherence tomography angiography classification of retinal neovascularization in retinopathy of prematurity
Source: Sci Rep. 2024 Jan 4;14:568. doi: 10.1038/s41598-023-49964-8 (PMC10766630; doi:10.1038/s41598-023-49964-8)
Supplement: Supplementary file 2 — Supplementary Table S1. [file 41598_2023_49964_MOESM2_ESM.pdf]

A pilot optical coherence tomography angiography classification of retinal neovascularization in retinopathy of prematurity

Xi Chen, Ryan Imperio, Christian Viehland, Pujan R. Patel, Du Tran-Viet, Shwetha Mangalesh, S. Grace Prakalapakorn, Sharon F. Freedman, Joseph A. Izatt, BabySTEPS group and Cynthia A. Toth

Supplementary Table 1. Demographics and Clinical Characteristics of the Included Subjects

	**TR-ROP group (7 infants)**	**Non-TR-ROP group (6 infants)**
**GA**	22 - 26 weeks	24 - 34 weeks
**Birth weight**	420 - 780 g	610 - 1415 g
**PMA at imaging**	32 – 47 weeks	36 - 49 weeks
**Ethnicity**	7/7 non-Hispanic	5/6 non-Hispanic
**Race**	3/7 black; 4/7 white	2/6 black; 4/6 white
**Sex**	5/7 male; 2/7 female	3/6 male; 3/6 female

*GA: gestational age; PMA: postmenstrual age; ROP: retinopathy of prematurity; TR-ROP: treatment-requiring ROP
